# Mindfulness and Compassion: An Examination of Mechanism and Scalability

**DOI:** 10.1371/journal.pone.0118221

**Published:** 2015-02-17

**Authors:** Daniel Lim, Paul Condon, David DeSteno

**Affiliations:** Department of Psychology, Northeastern University, Boston, Massachusetts, United States of America; Carnegie Mellon University, UNITED STATES

## Abstract

Emerging evidence suggests that meditation engenders prosocial behaviors meant to benefit others. However, the robustness, underlying mechanisms, and potential scalability of such effects remain open to question. The current experiment employed an ecologically valid situation that exposed participants to a person in visible pain. Following three-week, mobile-app based training courses in mindfulness meditation or cognitive skills (i.e., an active control condition), participants arrived at a lab individually to complete purported measures of cognitive ability. Upon entering a public waiting area outside the lab that contained three chairs, participants seated themselves in the last remaining unoccupied chair; confederates occupied the other two. As the participant sat and waited, a third confederate using crutches and a large walking boot entered the waiting area while displaying discomfort. Compassionate responding was assessed by whether participants gave up their seat to allow the uncomfortable confederate to sit, thereby relieving her pain. Participants’ levels of empathic accuracy was also assessed. As predicted, participants assigned to the mindfulness meditation condition gave up their seats more frequently than did those assigned to the active control group. In addition, empathic accuracy was not increased by mindfulness practice, suggesting that mindfulness-enhanced compassionate behavior does not stem from associated increases in the ability to decode the emotional experiences of others.

## Introduction

Meditation is now widely recognized to influence both functional and physiological aspects of the brain [1]. It has, for example, been shown to enhance attention [2], cognitive performance [3], [4], mental health [5],[6], and even cortical structure [7]. This long-standing emphasis on investigating the cognitive sequelae of meditation is in great part attributable to the assumption that meditation practice primarily targeted basic cognitive processes (see Davidson, 2010, for a review) [1]. In fact, one of the earliest and most basic styles of Buddhist meditation (i.e., *śamatha*) aims at improving concentration [8]. Nonetheless, it is also quite clear that, traditionally speaking, a primary goal of Buddhist meditation is an increase in virtuous mental states and behavior meant to counteract the causes of suffering (i.e., greed, hatred, and delusion) [9], [10].

In accord with this view, the effects of meditation on prosocial behavior have recently become a topic of scientific focus [11], [12], [13]. For example, Leiberg and colleagues demonstrated that an intensive one-day training of a compassion meditation technique enhanced prosocial economic decisions [12]. Similarly, Weng and colleagues [13] found that a two-week period of training in compassion meditation techniques enhanced altruistic financial behavior (i.e., accepting financial costs to oneself to benefit a victim of an unfair financial exchange). Moving beyond the financial realm, Condon and colleagues confirmed that participation in an eight-week course of mindfulness or compassion meditation significantly increased individuals’ willingness to come to the direct aid of another in pain [11]. Of import, the enhanced prosocial behavior meant to relieve the suffering of another occurred even in the face of a situation known to attenuate helping (i.e., the *bystander situation*; [14]).

These findings raise two important questions. The first centers on mechanism. Does meditation increase compassionate responding because it enhances empathic accuracy (i.e., the ability to correctly infer what another is feeling) or is there a different route? With respect to this question, the issue of whether empathic accuracy is required for meditation to enhance compassion has been raised by Klimecki and colleagues [15]. Although evidence exists that expert meditators show heightened neural responses to affective expressions of suffering during meditation [10], and that brief (i.e., 8-week) participation in compassion meditation training among novices can enhance emotion recognition [16], the role that such abilities may play in generating subsequent compassionate behavior remains unclear. For example, the ability to identify and simulate the pain of others might itself be maladaptive and constitute a source of burnout if experienced frequently [15]. Accordingly, the ability of mindfulness meditation to facilitate compassionate responding, outside of heightened empathic accuracy, might represent an alternative pathway for generating prosocial motivations.

The second question centers on scalability: Given that many individuals will not have easy access to meditation courses taught in person by expert practitioners or other certified teachers, can similar benefits be found using trainings created by such individuals that are delivered electronically via mobile devices? If so, the scalability of using meditation as a compassion intervention would appear promising, as individuals could practice at their convenience during daily life simply through utilizing a smartphone. Indeed, Weng et al. [13] utilized specially created audio segments supplied as compact discs or audio files as a primary tool for training in compassion meditation to good success. However, the use of such techniques to enhance behaviors directly targeting the relief of suffering within a face-to-face interpersonal context remains to be explored.

We designed the present experiment with these goals in mind. As such, we utilized a framework similar to that of our previous work [11], in which participants would take part in a brief course of meditation or not, and then be surreptitiously exposed to a situation which confronted them with the opportunity to relieve the pain of another. In addition, participants’ empathic accuracy would also be assessed to examine any meditation-based enhancements.

Although similar in structure to our previous work, the current experiment possesses three primary differences beyond the inclusion of a measure of empathic accuracy. The first involves the use of an active control group as opposed to a passive one. Given that the simple act of regular engagement in a task (as opposed to being assigned to a waiting list for a meditation course) might itself produce affective or motivational changes, control participants in the present experiment took part in a memory and cognitive skills training program. The second, as noted above, involves the use of a smartphone-based method of instruction (for both the meditation and control courses of instruction). In our previous work, a Buddhist lama delivered meditation instruction; here, meditation instruction is provided through the commercially available Headspace platform, which was designed by an individual with Buddhist monastic training. Finally, meditation instruction focuses solely on mindfulness meditation as opposed to compassion meditation. Many previous investigations of meditation on prosocial behavior have focused on compassion meditation. One potential problem with such strategies is the possibility of demand. Compassion training specifically emphasizes the importance of examining the feelings of others and behaving compassionately toward them, and as such, raises the possibility that ensuing effects on prosocial acts stem from demand characteristics.

Our previous work was the first to demonstrate that both compassion meditation and mindfulness meditation (which does not explicitly emphasize attention to the suffering of others) enhance prosocial behavior, while also utilizing a methodology specifically designed to reduce demand by unobtrusively measuring behaviors outside of a laboratory context [11]. Here, using a similar methodology, we focus solely on mindfulness techniques in order to examine the effects of mindfulness meditation on empathic accuracy and prosociality removed from concerns involving demand.

## Materials and Methods

### Participants

Sixty-nine participants with no previous experience practicing meditation were recruited from an undergraduate research pool at Northeastern University for a three-week experiment purported to be focused on mind training and cognitive skills. Given the longitudinal nature of the experiment, 6 individuals (9%) terminated participation prior to the experiment’s conclusion. We removed an additional 7 participants (11%) due to noncompliance with the experimental protocol. Noncompliant participants were defined as individuals who did not complete at least 12 out of the 14 meditation sessions. The final sample, therefore, consisted of 56 individuals (30 female, 26 male; mean age = 19.4 years, *SD* = 1.5), all of whom reported little to no prior experience with meditation. Participants received credit as part of an introductory psychology course requirement for their participation. In addition, we informed participants that they would be entered in a lottery for a $100 Amazon.com gift voucher to be awarded at completion of the entire study.

### Procedure

Participants were randomly assigned to one of two experimental conditions: (1) a three-week mindfulness-based meditation condition that involved regularly completing a meditation session using a self-administered and self-guided web-based application (*Headspace*, www.getsomeheadspace.com), or (2) an active control group which involved the use of a three-week web-based cognitive training program that was also self-administered and self-guided (*Lumosity*, www.lumosity.com).

The experiment unfolded in three phases: (1) *briefing*, (2) *training period*, and (3) *in-lab experiment*.

***Briefing***. Prior to the start of the training program, participants attended a briefing session in which written informed consent was obtained. In addition, participants were screened for any previous experiences with meditation. Upon completion of the screening questionnaire, participants were briefed on the protocol they would follow which included a demonstration of the online web and/or mobile interface of the training programs to which they were assigned. Participants were then issued a pre-registered account for their assigned training program.

***Training Period***. Participants were instructed to complete 14 training sessions (i.e., *Headspace* or *Lumosity* depending on condition) within a three-week period. As the participants were assigned a pre-registered account on either Lumosity or Headspace, the research staff had access to the participants’ account during the training period, thereby allowing the record of each participant’s progress to be monitored on a daily basis (i.e., whether or not a participant had completed a training session for any given day in the three-week training period). In addition, participants in the mindfulness-based meditation condition (*Headspace*) were also instructed to complete a daily quiz online following each of the 14 sessions to test for their comprehension of the instructional content of the online training program. Similarly, participants in the active control condition (*Lumosity*) were asked to complete a daily questionnaire online that probed their experience during the training session (e.g. How tired were you during the session? How attentive were you during the session?). These measures allowed us to monitor continued engagement remotely. Reminder emails were sent to participants during the first and second week of the training period to ensure compliance with the study’s protocol.

Participants who were assigned to the Headspace training program were instructed to complete mindfulness-based meditation sessions designed by an individual with Buddhist monastic training from the “Take 10” and “Take 15” meditation program (10 days of “Take 10” and first 4 days of “Take 15”, with a total of 14 days of training). Each training session averaged 12 minutes a day. The training program centered on mindfulness meditation, which included focusing on a selected object (e.g. the body or the breath), monitoring the activity of the mind, noticing mind-wandering, and developing a non-judgmental orientation toward one’s experience (i.e., equanimity). The training did not include any references to loving-kindness, compassion, or related terms. The training application was designed to allow participants to complete multiple sessions on the same day if they chose to; therefore, participants were explicitly instructed to complete only one session per day. Furthermore, research assistants monitored the participant’s daily progress to ensure that participants did not complete multiple sessions in a given day.

Participants assigned to the Lumosity training program were required to complete mind-training sessions (14 days in total). Each session consisted of a series of 5 mini-games that were related to memory, attention, speed, flexibility, and problem solving. Each session took approximately 10 minutes to complete. As free Lumosity accounts were utilized, participants were limited to only 1 training session per day; therefore, there was no risk of participants completing multiple sessions in a single day.

***Lab Experiment***. Upon the completion of the training program, participants returned to the lab one at a time for a post-training test session. While sitting in the waiting area outside the lab, participants were confronted with a task designed to assess compassionate responding [11]. Participants were then invited into the lab and completed the Emotion Recognition Index [17] as a measure of empathic accuracy.

***Ethics Statement***. The protocol for this experiment was approved by the Northeastern University Institutional Review Board (approval number: 13–11–02; approved November 21, 2013). Participants were debriefed via email once the entire study had ended, so as to prevent any suspicion from arising among participants with regards to the behavioral paradigm for measuring compassionate responding.

### Measures

***Compassionate Responding***. To measure compassionate responding, we utilized a naturalistic and ecologically valid behavioral measure developed by Condon and colleagues [11]. This behavioral paradigm utilizes a staged scenario with three confederates. As participants (all of whom completed this task individually) entered a common waiting area for many labs in a public hallway, they encountered a row of three chairs. Two male confederates occupied two of the chairs, leaving one remaining seat for the participant. After a participant had been sitting for 1 minute, a female confederate, playing the role of the “sufferer,” appeared from around the corner down the hallway with the use of a large walking boot and a pair of crutches. She walked with some difficulty and expressions of mild pain (i.e., wincing). Upon arriving in the waiting area, the suffering confederate stopped beside the seat furthest away from the participant, let out an audible sigh of discomfort, and leaned against the wall as if she were also waiting for an experiment. The male confederates were trained to act indifferent to the female confederate (i.e., ignore her). Immediately after the suffering confederate leaned against the wall in her final position, one of the sitting confederates started a timer. This situated ended following one of two outcomes: (1) the true participant offered his or her seat to the confederate on crutches, or (2) two minutes passed without any helping response. Compassionate responding was coded as a dichotomous variable: participants gave up their seat to the confederate on crutches or not [11].

In our previous work [11], the sitting confederates were always female. In the present study, we included male confederates to further examine the generalizability of our previously reported effect of mindfulness- and compassion-based meditation on compassionate responding. It remains to be explored how the nature of the effect might change if a male confederate played the role of the suffering confederate.

***Empathic Accuracy***. As a performance-based measure of empathic accuracy, we utilized the Emotion Recognition Index [17]. It consists of 60 test items which are divided into 2 subtests: (1) a facial subtest that utilizes 30 photographs from the Pictures of Facial Affect (POFA) [18] as test items for measuring accuracy in facial emotion recognition, and (2) a vocal subtest that employs 30 non-verbal recordings from the International Study of Vocal Emotion Expression [19] as test items for measuring accuracy affective vocal recognition. Scores range from 0 (complete inaccuracy) to 1 (complete accuracy) on a continuous scale. The advantage of using the ERI over other tests of empathic accuracy (e.g. Reading the Mind in the Eyes Test [20]) is that it allows the user to measure empathic accuracy over two channels: (1) the vocal channel (via the vocal subtest) and the (2) the visual channel (via the facial subtest).

## Results

In accord with predictions, participants who underwent a three-week mindfulness-based meditation program evidenced enhanced compassion. They were significantly more likely to give up their seats (37%) to the suffering confederate compared with those in the active control group (14%), *χ* ^2^ (1, *N* = 56) = 4.03, *p* < .05, *ϕ* = .27 (see Table 1). Of import, the relative level of compassionate behavior in the active control group virtually matched that of the passive control group (16%) from Condon et al.[11], providing additional assurance that the 23% increase in helping among meditating participants truly represents an increase from baseline (i.e, as opposed to stemming from the active control training somehow producing a decrease in what would have been the normative level of compassionate responding). Also consistent with Condon et al. [11], analysis revealed that there were no gender differences in compassionate responding, *χ* ^2^ (1, *N* = 56) = 2.39, *p* = .15.

**Table 1:** Observed and Expected Frequencies for Helping Outcome Between Conditions.

	Meditation (Headspace)		Active Control (Lumosity)	
Outcome	Observed	Expected	Observed	Expected
Helped	10	6.25	4	7.25
Did not Help	17	20.75	25	21.75

We next examined whether mindfulness training enhanced empathic accuracy by subjecting participants’ scores on the ERI to a one-way ANOVA. Supporting the view that mindfulness-enhanced compassion need not stem from heightened empathic accuracy, participants in the mindfulness and control conditions demonstrated equivalent scores on the ERI (Lumosity: *M* = 0.68, *SD* = .05; Headspace: *M* = 0.68, *SD* = .07). Participants also did not differ in their scores on the vocal subtest (Lumosity: *M* = 0.66, *SD* = .08; Headspace: *M* = 0.66, *SD* = .09) and the facial subtest (Lumosity: *M* = 0.71, *SD* = .06; Headspace: *M* = 0.69, *SD* = .08) of the ERI, all *t’s* < 1.

## Discussion

These findings are notable for several reasons. First, they serve as a robust replication of our earlier findings demonstrating that brief engagement in mindfulness meditation enhances compassionate behavior [11]. Second, they confirm that such enhanced prosocial behavior need not stem from alterations in empathic accuracy. That is, increased motivations to relieve the suffering of others did not stem from a concomitant sharpening of skills in reading the emotions of others [15]. It is important to note, however, that our findings do not preclude the possibility that continued training in meditation might alter empathic abilities [10], [16]; rather, they simply show that any such increases need not always underlie greater compassionate behavior. It is important to note, however, that the present findings do not preclude the possibility that mindfulness and compassion-based meditation might increase compassionate outcomes via different mechanisms. Whereas compassion meditation might increase compassionate behaviors through empathic processes and prosocial emotion, mindfulness-meditation might increase compassionate behaviors through a number of plausible mechanisms, including increased attention to all stimuli [21] or a reduction of self-related affective biases [22], [23]. Future work should prioritize examination of practice-specific mediators of enhanced compassionate behavior in order to determine whether different practices are more or less effective for promoting compassionate outcomes for specific populations.

These findings also point to the potential scalability of meditation as a technique for building a more compassionate society. As is clearly evident, many individuals do not have the luxury of time or accessibility to regularly attend meditation training sessions with certified instructors. The ability to access such expert guidance using web- and mobile-based technology at little cost would greatly facilitate engagement in contemplative practice among any interested individuals. Moreover, the potential for the rapid spread of prosocial behavior would be strengthened not only by the increased numbers of individuals demonstrating increased compassionate motivations, but also by a “pay-it-forward” effect among recipients of their kindness. As our past work has shown, grateful beneficiaries of aid evidence a marked increase in their likelihood to subsequently extend help to others, even if these others are complete strangers [24], [25]. Accordingly, the potential for efficient, fairly rapid deployment of mindfulness-based benefits on compassionate responding appears worthy of increased investigation. Of note, however, such investigation need also consider the potential of deleterious side effects that might arise from extended meditation practice that does not include the ability for dynamic, personalized give-and-take between an instructor and pupil (e.g., monitoring for mental health issues that might arise from failures in emotional regulation or anxiety brought on by examining existential concerns).

## Supporting Information

S1 Data Data File. (XLSX)

S1 File Codebook for Data File. (DOCX)
