# Supplementary material for: Mindfulness and Compassion: An Examination of Mechanism and Scalability
Source: PLoS One. 2015 Feb 17;10(2):e0118221. doi: 10.1371/journal.pone.0118221 (PMC4331532; doi:10.1371/journal.pone.0118221)
Supplement: S1 File — (DOCX) [file pone.0118221.s002.docx]

**Variable Coding:**

**ID**: Randomly generated alphanumeric identification code for the participants.

**Condition**: Experimental condition assigned to the participants. 1 = Headspace (mindfulness meditation condition), 2 = Lumosity (active control group).

**Helped**: Observed helping behavior of the participants (whether or not they gave up their seat to the confederate in crutches). 1 = Yes, 0 = No.

**ERIFace**: The participants’ total score on the facial subtest of the Emotion Recognition Index. The score is a function of the proportion of items that were answered correctly. Scores were calculated using the SPSS Syntax provided in the test package.

**ERIVocal**: The participants’ total score on the vocal subtest of the Emotion Recognition Index. The score is a function of the proportion of items that were answered correctly. Scores were calculated using the SPSS Syntax provided in the test package.

**ERITotal**: The participants’ grand total score on the Emotion Recognition Index. The score is a function of the proportion of items that were answered correctly. Scores were calculated using the SPSS Syntax provided in the test package.

**Gender**: The participant’s gender. 1 = Female, 2 = Male.
